# Correlation analysis between hemoglobin and type 2 diabetic nephropathy: a two-center retrospective study

**DOI:** 10.1007/s00592-025-02529-9

**Published:** 2025-06-05

**Authors:** Xiaoling Liu, Ze Zhang, Lu Lin, Jinghui Li, Bende Liu, Xiangjin Xu, Huaqian Chen, Junwei Zhou, Pin Chen

**Affiliations:** 1 Fuzong Clinical Medical College of Fujian Medical University, 900th Hospital of PLA Joint Logistic Support Force, Fuzhou, China; 2 Department of Nephrology, Sinopharm Dongfeng General Hospital, Hubei University of Medicine, Shiyan, China; 3 Department of Emergency Medicine, Union Hospital, Tongji Medical College, Huazhong University of Science and Technology, Wuhan, China; 4 Department of Endocrinology, Fuzong Clinical Medical College of Fujian Medical University, 900th Hospital of the Joint Logistics Team, Fuzhou, China

**Keywords:** Hemoglobin, Type 2 diabetes mellitus, Diabetic nephropathy, Biomarker

## Abstract

**Objective:**

This study aimed to investigate the relationship between hemoglobin (Hb) levels and diabetic nephropathy (DN) in patients with type 2 diabetes mellitus (T2DM).

**Methods:**

We recruited patients with T2DM from two centers, collected their clinical data, and analyzed the relationship between Hb levels and DN using binary logistic regression analysis and restricted cubic spline squares (RCS) plots.

**Results:**

A total of 1956 patients with T2DM were enrolled, including 784 (40.1%) with DN. After adjusting for several confounding factors, the proportion of patients with DN in Q1 of Hb was significantly higher than that in the other group. The RCS curve revealed that Hb levels were inversely proportional to DN. Subgroup analysis demonstrated that age, gender, history of hypertension, history of nonalcoholic fatty liver disease and history of diabetic retinopathy exhibited no significant correlation with Hb levels and DN.

**Conclusions:**

Low Hb level is closely associated with DN occurrence, and can be used as a predictive biomarker for DN.

**Supplementary Information:**

The online version contains supplementary material available at 10.1007/s00592-025-02529-9.

## Introduction

According to the International Diabetes Federation, the global prevalence of diabetes mellitus was approximately 537 million in 2021, which will continue to increase in the coming years [1]. In addition to hyperglycemia, patients with diabetes may develop macroangiopathy, microangiopathy and neuropathy. Diabetic nephropathy (DN) is one of the most common microvascular complications of diabetes mellitus, occurring in approximately 40–50% of patients with diabetes [2].The incidence of DN is slightly higher in patients with type 2 diabetes mellitus(T2DM) than in those with type 1 diabetes mellitus. The key clinical manifestations of DN include a decrease in the estimated glomerular filtration rate (eGFR) and an increase in urinary protein, leading to renal failure and severely affecting the quality of life and health of patients [3].DN is one of the leading causes of end-stage renal disease, posing a significant economic and medical burden. DN pathogenesis is primarily related to the accumulation of glycation products, insulin resistance, oxidative stress, inflammatory response and other mechanisms. Additionally, impaired endothelial cell function is an important factor in DN progression [4, 5]. Studies have reported that the deformability of red blood cells may be one of the mechanisms underlying diabetic microvascular complications, which is critical in the progression of DN and diabetic foot. Progressive impairment of red blood cell deformability is associated with the loss of renal function in patients with diabetes, and changes in hemoglobin (Hb) levels are essential for red blood cell deformability [6, 7]. Although decreased renal function can lead to anemia in patients with diabetes, there is evidence that the incidence of anemia is significantly higher in patients with diabetes with normal renal function [8]. Furthermore, Hb is associated with adverse outcomes in kidney disease, including deterioration of kidney function, cardiovascular events and fractures [9, 10]. However, few studies have examined the correlation between Hb level and DN. This study aimed to analyze the correlation between Hb level and DN and its relationship with DN severity using a two-center cross-sectional design.

## Methods

### Population

We screened patients with T2DM admitted to the Department of Endocrinology of the 900 Hospital of the Joint Logistics Team and the Sinopharm Dongfeng General Hospital between January 2018 and December 2023. The inclusion criteria were as follows: Diagnosed with T2DM, age ≥ 18 years, and complete clinical data and laboratory test results [11]. The exclusion criteria included the following: Secondary diabetes mellitus, primary nephrotic syndrome, glomerulonephritis and secondary nephrotic disease like systemic lupus erythematosus, as well as acute complications of diabetes mellitus, infection and severe heart and lung diseases. DN was diagnosed on based on urinary albumin/creatinine ratio (UACR) and eGFR [12]. This retrospective study was approved by the Medical Ethics Committee of our hospital (Ethics No.:2023-055). This study followed the 1964 Declaration of Helsinki and informed consent was obtained from the patients.

## Data collection

General patient data were collected, including age, sex, height, weight, systolic blood pressure (SBP), diastolic blood pressure (DBP), history of nonalcoholic fatty liver disease (NAFLD), history of coronary atherosclerotic heart disease (CHD), diabetic peripheral neuropathy (DPN), diabetic peripheral vascular disease (DPV), diabetic retinopathy (DR), and DN.

Laboratory data were collected, including: white blood cell (WBC), neutrophil (NE), red blood cell (RBC), Hb, urea nitrogen (BUN), creatinine (CR), uric acid (UA), alanine aminotransferase (ALT), aspartate aminotransferase (AST), total protein (TP), albumin (Alb), total cholesterol (TC), triglyceride (TG), high-density lipoprotein (HDL), low-density lipoprotein (LDL), glycosylated hemoglobin (HbA1c), and UACR. *As UACR is not necessary for some patients with end-stage renal disease*,* we only discussed the predictive power of UACR in patients with CKD1-4 in this study.*

The eGFR (Chronic Kidney Disease Epidemiology Collaboration, CKD-EPI) was calculated using the following equation:

eGFR = 142 × (Scr/A)^B^ × (0.9938)^age^ × C (Scr: serum creatinine (mg/dL)). (1) Female: C = 1.012, Scr ≤ 0.7 mg/dL, A = 0.7, B = − 0.241; Scr > 0.7 mg/dL, A = 0.7, B = − 1.2. (2) Male: C = 1, Scr ≤ 0.9 mg/dL, A = 0.9, B = − 0.202; Scr > 0.9 mg/dL, A = 0.9, B = − 1.2.

### Statistical analysis

Continuous variables are expressed as mean (standard deviation). The independent sam*p*le t-test was used for normally distributed data, and the Mann–Whitney U test was used for non-normally distributed data. Categorical variables are expressed as frequencies (percentages), and the chi-square test was used for statistical analysis.

Hb was divided into quartiles (Q1, Q2, Q3, and Q4), and the association between Hb and DN was evaluated using multivariate logistic regression analysis (LRA), adjusting for possible confounding factors, including age and gender. Restricted cubic spline (RCS) curves were used to investigate the nonlinear relationship between Hb level and DN. To evaluate the association between Hb and DN, subgroup analysis was performed based on gender, age, HbA1c > 7%, history of hypertension, history of NAFLD, and DR. The interaction between these variables and Hb was determined using multivariate LRA.

## Results

### Baseline characteristics of all patients

**Fig. 1:**
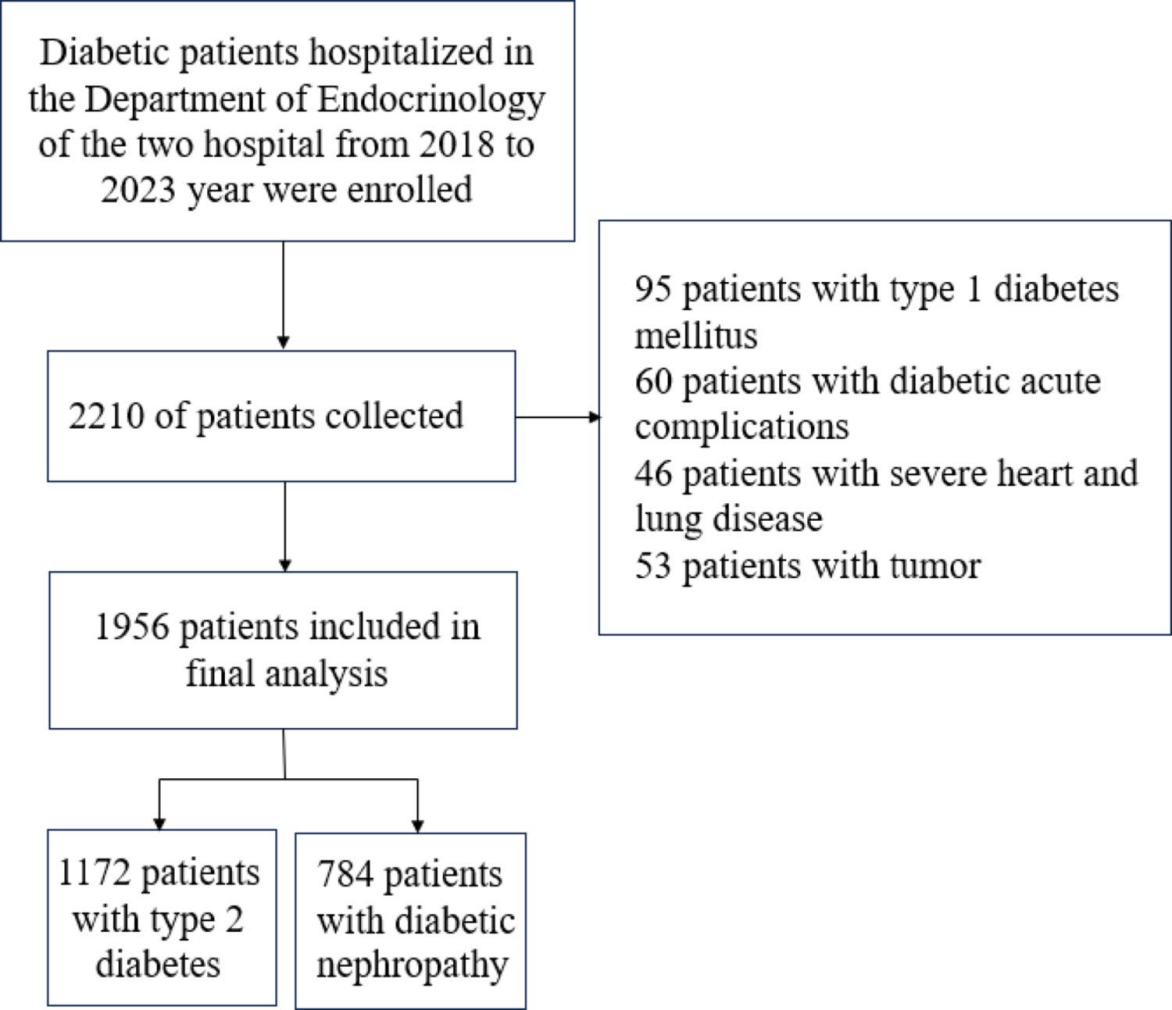
Flow chart for the screening of enrolled patients

A total of 1956 patients with T2DM (mean age 59.41 ± 13.22 years) were enrolled from two centers, including 1172 patients without DN and 784 with DN. Comparison of the baseline data of these patients revealed significant differences in age, height, weight, SBP, DBP, WBC, RBC, Hb, BUN, CR, Alb, HDL, LDL, history of hypertension and NAFLD, DPN, DPV, DR, CHD, eGFR and UACR between DN and T2DM groups (*P* < 0.05) (Fig. 1; Table 1).

**Table 1:** The baseline data of the two groups were compared

	Overall	T2DM	DN	*p*
n	1956	1172	784	
age (years)	59.41 (13.22)	57.31 (13.66)	62.54 (11.86)	< 0.001
height (cm)	165.12 (8.39)	165.61 (8.82)	164.38 (7.63)	0.001
weight (kg)	65.65 (14.73)	67.17 (12.83)	63.37 (16.93)	< 0.001
SBP (mmHg)	129.40 (17.92)	125.92 (15.77)	137.90 (19.93)	< 0.001
DBP (mmHg)	76.63 (10.95)	76.20 (10.78)	77.68 (11.29)	0.015
WBC (*10^9/L)	6.82 (2.12)	6.69 (1.88)	7.01 (2.42)	0.001
NE (*10^9/L)	4.31 (1.89)	3.98 (1.60)	4.79 (2.17)	< 0.001
RBC (*10^9/L)	4.16 (0.86)	4.57 (0.57)	3.55 (0.87)	< 0.001
Hb (g/L)	122.99 (26.42)	135.86 (17.18)	103.74 (26.12)	< 0.001
BUN (mmol/L)	9.65 (8.29)	5.39 (1.70)	16.02 (9.97)	< 0.001
CR (µmol/L)	205.36 (270.48)	67.85 (16.92)	410.93 (334.09)	< 0.001
UA (µmol/L)	353.17 (116.00)	317.20 (99.22)	406.94 (118.49)	< 0.001
ALT (U/L)	27.58 (85.60)	28.07 (29.67)	26.85 (130.29)	0.758
AST (U/L)	24.54 (122.63)	22.30 (23.49)	27.90 (191.58)	0.323
TP (g/L)	67.17 (7.85)	68.40 (7.09)	65.32 (8.54)	< 0.001
Alb (g/L)	39.51 (5.71)	41.32 (4.57)	36.79 (6.15)	< 0.001
TC (mmol/L)	4.75 (1.51)	4.78 (1.48)	4.69 (1.55)	0.198
TG (mmol/L)	2.37 (3.14)	2.40 (3.71)	2.33 (2.00)	0.647
HDL (mmol/L)	1.08 (0.34)	1.11 (0.34)	1.04 (0.33)	< 0.001
LDL (mmol/L)	2.99 (1.10)	3.01 (1.02)	2.95 (1.19)	0.192
HbA1c (%)	9.68(2.48)	9.82(2.4)	9.25(2.45)	< 0.001
Hypertension (%)				< 0.001
no	780 (39.9)	668 (57.0)	112 (14.3)	
yes	1176 (60.1)	504 (43.0)	672 (85.7)	
NAFLD (%)				< 0.001
no	1284 (65.6)	631 (53.8)	653 (83.3)	
yes	672 (34.4)	541 (46.2)	131 (16.7)	
DPN (%)				< 0.001
no	1443 (73.8)	830 (70.8)	613 (78.2)	
yes	513 (26.2)	342 (29.2)	171 (21.8)	
DPV (%)				0.01
no	1583 (80.9)	926 (79.0)	657 (83.8)	
yes	373 (19.1)	246 (21.0)	127 (16.2)	
DR (%)				< 0.001
no	1663 (85.0)	1064 (90.8)	599 (76.4)	
yes	293 (15.0)	108 (9.2)	185 (23.6)	
CHD (%)				< 0.001
no	1611 (82.4)	1038 (88.6)	573 (73.1)	
yes	345 (17.6)	134 (11.4)	211 (26.9)	
sex (%)				0.994
female	700 (35.8)	420 (35.8)	280 (35.7)	
male	1256 (64.2)	752 (64.2)	504 (64.3)	
eGFR	92.97(27.35)	102.89(16.57)	62.39(31.17)	< 0.001
UACR	40.68(43.02)	1.02(2.06)	32.71(206.64)	< 0.001

Abbreviations: SBP, systolic blood pressure; DBP, diastolic pressure; WBC, white blood cell; NE, neutrophil; RBC, red blood cell; Hb, hemoglobin; BUN, urea nitrogen; CR, creatinine; UA, uric Acid; ALT, alanine aminotransferase; AST, aspartate aminotransferase; TP, total protein; Alb, albumin; TC, total cholesterol; TG, triglyceride; HDL, high-density lipoprotein; LDL, low-density lipoprotein; NAFLD, non-alcoholic fatty liver disease; DPN, diabetic peripheral neuropathy; DPV, diabetic peripheral vascular disease; DR, diabetic retinopathy; CHD, coronary heart disease; eGFR, Estimated glomerular filtration rate; UACR, urine albumin creatinine ratio

### Correlation between hb and DN

**Fig. 2:**
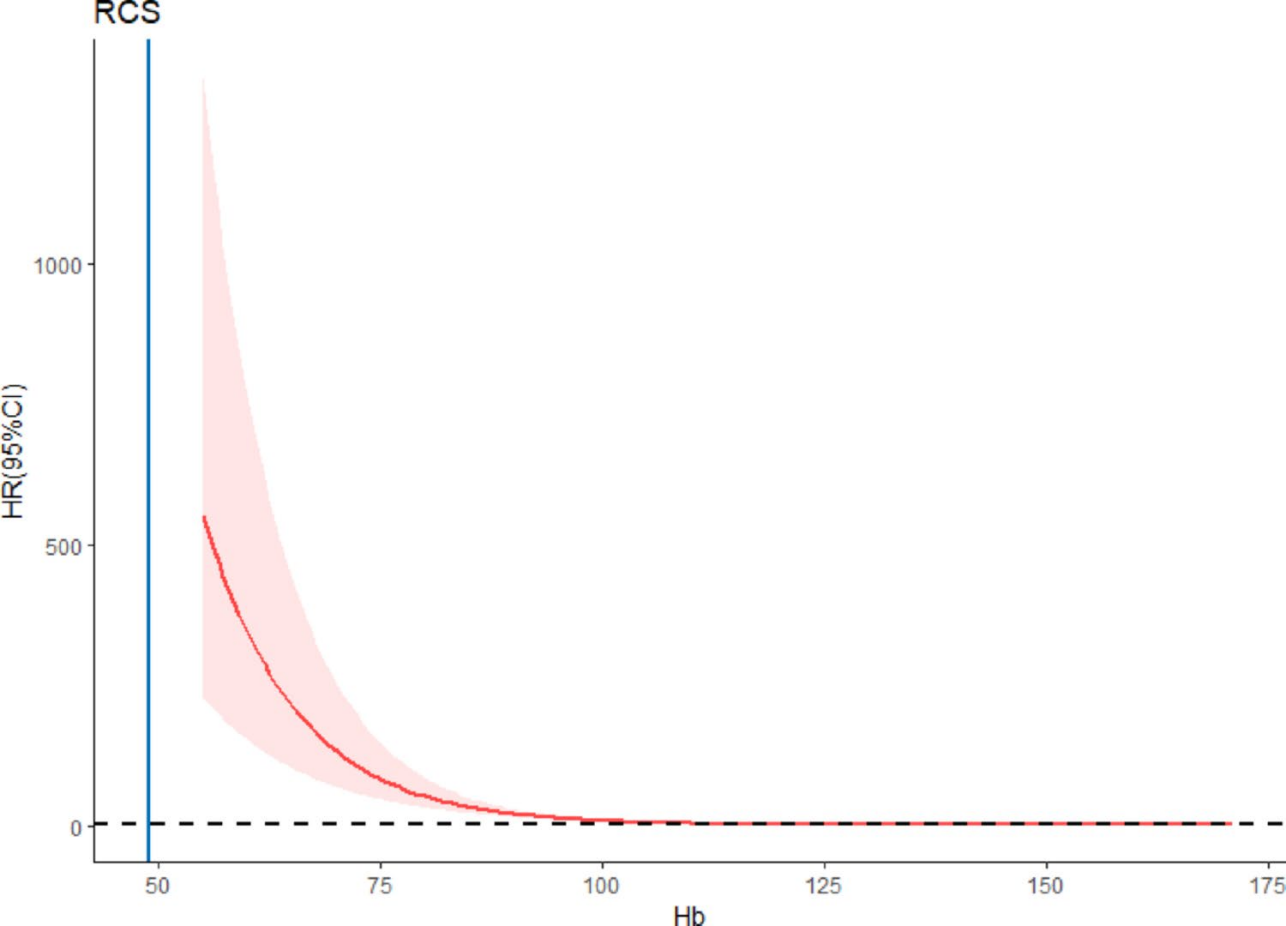
RCS curve of the correlation between Hb level and DN

**Fig. 3:**
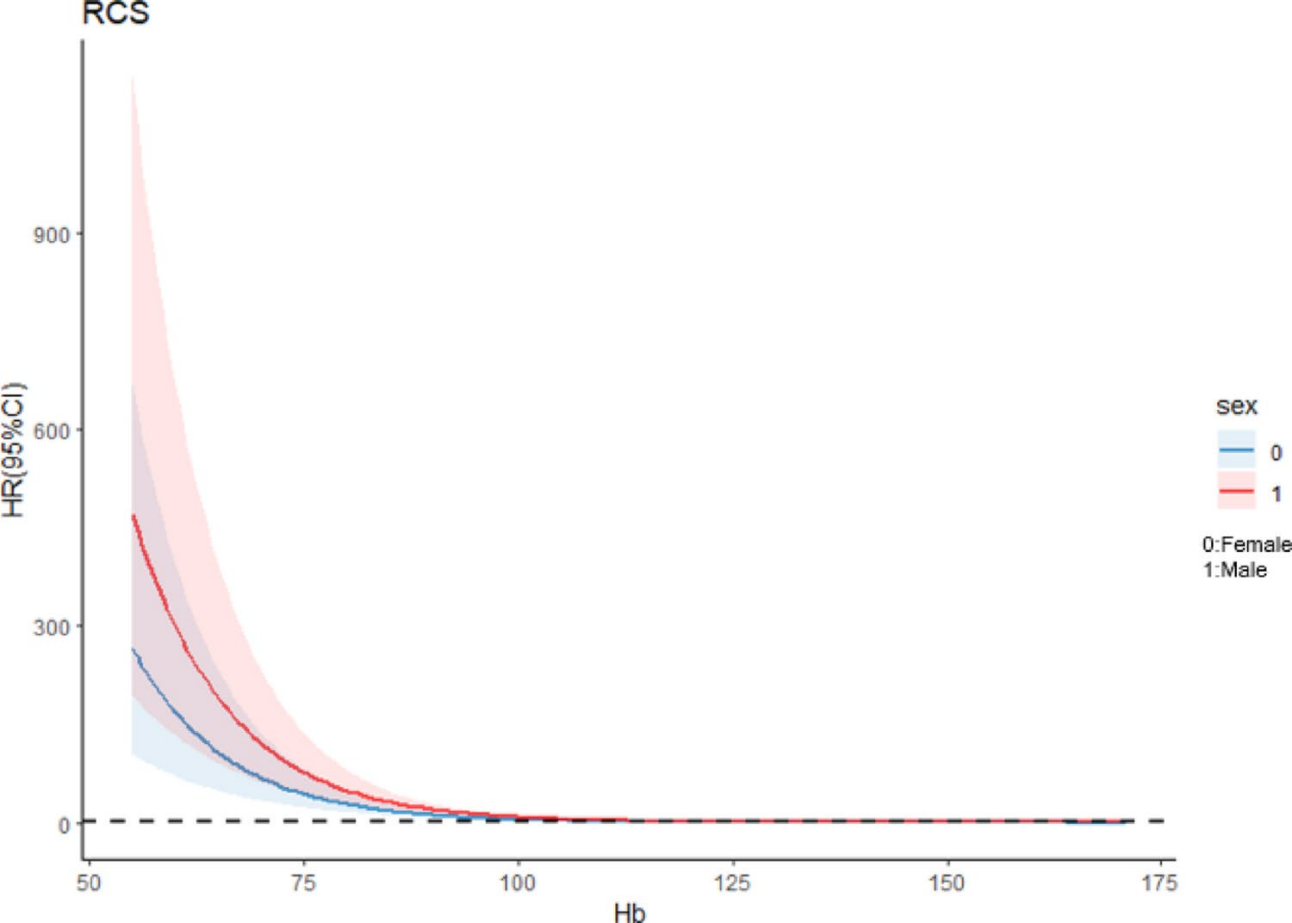
RCS curves of the association between Hb level and DN stratified by sex

The quartile (Q1, Q2, Q3, and Q4) ranges of Hb were < 106 g/L, 106–128 g/L, 128–143 g/L, and > 143 g/L. After adjusting for confounding factors, including age, sex, SBP, DBP, height, weight, WBC, NE, TC, TG, HDL, and LDL, a significant correlation was observed between Hb level and DN. When Q1 of Hb was used as a reference, Q4 exhibited the smallest OR value (0.270 [0.159–0.453]; *P* < 0.05; Table 2). Additionally, RCS plots revealed an inverse relationship between Hb levels and DN. The inflection point value was 128, indicating that the correlation trend between DN and Hb increased significantly when the Hb level was < 128 g/L. When gender was used as a subgroup, the RCS curve revealed that the Hb level was inversely proportional to DN (Figs. 2 and 3).

**Table 2:** Relations between hemoglobin and diabetic kidney disease in patients with DN

	Hb				
	Q1(406)	Q2(397)	Q3(390)	Q4(359)	*P* value for trend
model1 OR (95%CI)	Ref	0.238(0.17,2-0.327)	0.146(0.100-0.211)	0.162(0.107–0.240)	< 0.001
model2 OR (95%CI)	Ref	0.254(0.181–0.354)	0.147(0;099-0.217)	0.173(0.112–0.264)	< 0.001
model3 OR (95%CI)	Ref	0.357(0.238–0.532)	0.255(0.160–0.401)	0.245(0.145–0.407)	< 0.001
model4 OR (95%CI)	Ref	0.367(0.244–0.549)	0.277(0.173–0.440)	0.270(0.159–0.453)	< 0.001

The quartile ranges of Q1, Q2, Q3, Q4 of Hb were < 106,106–128,128–143,>143. Model1 was adjusted for sex and age. Model 2 was further adjusted for diastolic blood pressure, systolic blood pressure, height and body weight. Model 3 was further adjusted for WBC, NE, PLT, BUN. Model 4 was further adjusted for TC, TG, HDL, LDL. Q1 is the reference group. Multivariate logistic regression analyses were performed to estimate the ORs and corresponding 95% CIs for diabetic kidney disease. Abbreviations: Q: quartile, OR: odds ratio, CI: confidence interval, Hb: hemoglobin

The analysis of the distribution of the Hb quartile in T2DM and DN groups revealed that the proportion of Hb in the Q1 stage was highest in the DN group and lowest in the T2DM group. Moreover, patients with CKD stage 5 exhibited the highest proportion of patients Q1 of Hb and the lowest in Q4 of Hb (Table 3a and b). *We compared the diagnostic value of Hb with the traditional DN biomarkers UACR and eGFR. Their AUC and 95% confidence interval (CI) were 0.624 (0.593–0.654)*,* 0.809(0.790–0.829)*,* and 0.604 (0.573–0.634)*,* respectively. The diagnostic efficacy of UACR was the highest*,* followed by Hb*,* while eGFR exhibited slightly higher predictive sensitivity than Hb but lower specificity* (Table 4).

**Table 3:** a. Proportion of patients in the two groups within each interval of the hb quartile

	Hb-Q1	Hb-Q2	Hb-Q3	Hb-Q4	*P*
T2DM	5%	24.80%	36.80%	33.40%	< 0.001
DN	55.60%	25.40%	11.10%	7.90%	

**Table 3:** B proportion of patients with different stages of DN in HB quartile interval

	Hb-Q1	Hb-Q2	Hb-Q3	Hb-Q4	*p*
CKD1	3.60%	20.90%	37.10%	38.40%	< 0.001
CKD2	12.80%	42.80%	31.60%	12.80%	
CKD3	32.50%	41.90%	17.50%	8.10%	
CKD4	61.40%	35.10%	3.50%	0.00%	
CKD5	84.20%	13.50%	1.50%	0.80%	

**Table 4:** Comparison of hb and eGFR in DN diagnosis

	AUC	95%CI	Cut-off	sensitivity	Specificity
eGFR	0.604	0.573–0.634	0.234	94.10%	24.40%
UACR	0.809	0.790–0.829	0.306	85.50%	69.10%
Hb	0.624	0.593–0.654	0.317	66.20%	51.80%

## Subgroup analysis

**Fig. 4:**
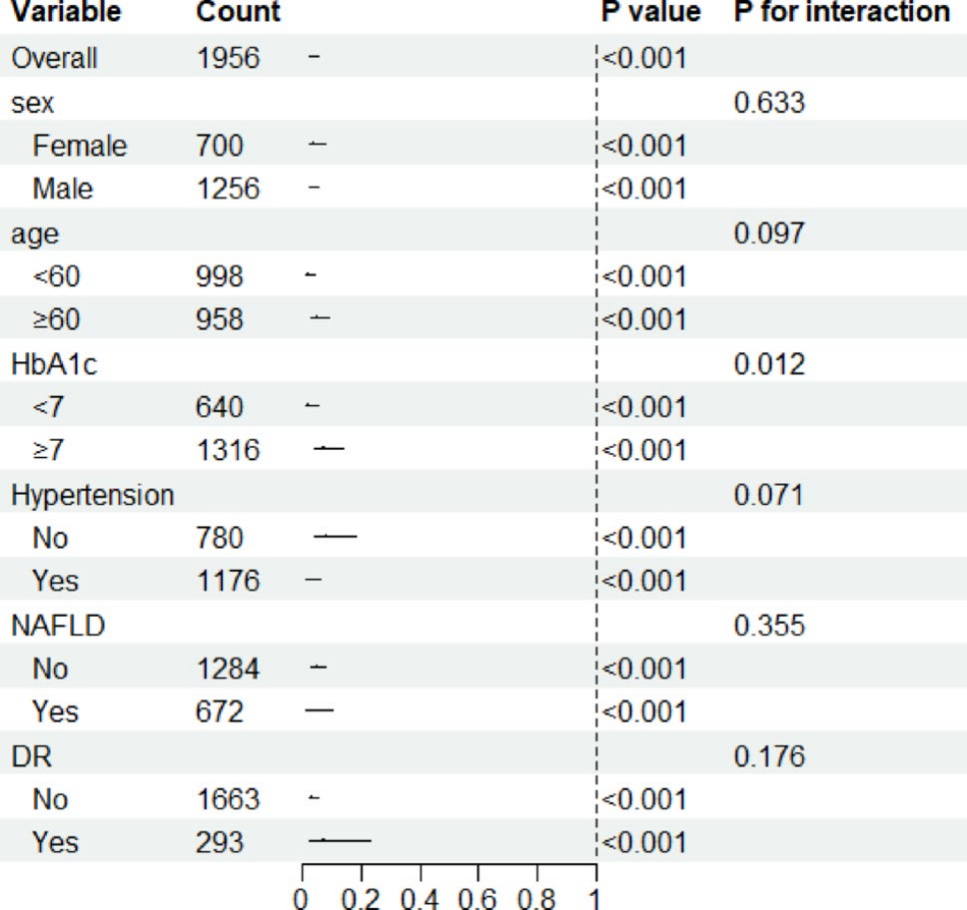
Forest plots of the subgroup analyses

To investigate the correlation between Hb and DN in different populations, the patients were grouped according to sex, age (≥ 60 years), HbA1c (≥ 7%), history of hypertension, history of NAFLD, and diabetic retinopathy. These results demonstrated that Hb levels were negatively correlated with DN. Other factors exhibited no significant correlations with Hb levels (Fig. 4).

**Fig. 5:**
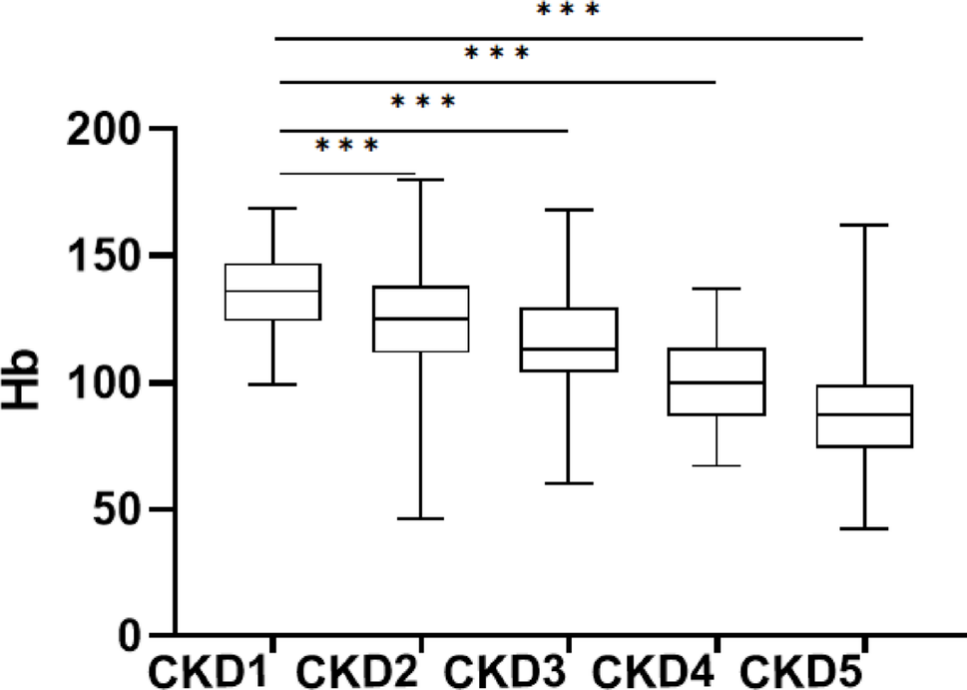
Hb level of patients with different CKD stages

To investigate the relationship between Hb levels and different DN stages, all DN patients were classified into five stages based on eGFR. Compared with the Hb levels of patients at different stages, kidney function gradually deteriorated with a decrease in the Hb levels (Fig. 5).

## Discussion

DN is a common complication of diabetes mellitus. Early diagnosis of DN is crucial for disease assessment and early intervention. Our results revealed a significant correlation between Hb and DN, with the incidence of DN increasing significantly as Hb levels decreased. When Hb was < 106 g/L, the prevalence of DN and the risk of progression to clinical renal disease stage were significantly increased. With an increase in Hb levels, the prevalence of DN gradually decreased, and the stage of DN tended to be lower. Consequently, the Hb level could be valuable for the diagnosis of DN. Although the relationship between Hb and DN has been previously reported, we analyzed the Hb levels of patients with diabetes in two research centers with a larger sample size, which could better reflect the association between Hb and DN [13–15].

The occurrence of diabetic microvascular complications depends on changes in microvascular hemodynamics and oxygen delivery system. When the deformability of red blood cells decreases, their osmotic fragility declines, the oxygen dissociation curve shifts to the right, and the shear stress increases, thereby increasing the risk of microvascular injury [16]. Hyperglycemia in T2DM has multiple effects on red blood cells, including changes in cell deformability and increased adhesion, increased Hb glycation, increased red blood cell distribution width and decreased Hb levels in diabetic patients [17]. Impaired erythropoietin production leads to anemia in the end-stage of DN and CKD. Moreover, the baseline Hb level affects renal outcomes, possibly due to anemia causing capillary hypoperfusion and imbalances in cellular oxygen delivery and energy metabolism. Several previous small- sample studies have reported that the baseline Hb level in patients is correlated with DN progression and can be used as a predictor of renal fibrosis [18]. In a clinical study, Zou et al. [19] used machine learning algorithms to construct a prediction model for end-stage renal disease, in which Hb was an important factor. The results of a cohort study revealed that Hb level was negatively correlated with the severity of renal pathological changes in patients with DN, indicating that Hb concentration could reflect early renal fibrosis and tubular injury and serve as a risk factor for DN [20]. Hb was an independent risk factor for developing renal failure in DN (OR = 0.97; 95% CI = 0.948–0.990), and could be used as a predictor of renal function deterioration [21]. Our results demonstrated that Hb levels were associated with DN development even when Hb levels were within the normal range, consistent with the results of another cohort study [22].

Epidemiological surveys have reported that most patients with diabetes are iron deficient, with a high prevalence of anemia [23]. Hb can indirectly reflect the redox state of the body because approximately 3/2 of the iron in the body is bound to Hb. Iron ions are released when oxygen enters the cells from the lungs, thereby affecting the process. The accumulation of iron ions in the body leads to oxidative stress and abnormal insulin synthesis and release, thereby increasing the risk of diabetes [24]. Furthermore, the Hb level is associated with the outcomes of end-stage renal disease, including cardiovascular events and mortality [25]. A decrease in Hb level may lead to endothelial cell dysfunction, thereby increasing capillary permeability and accelerating DN progression [5]. Endothelial dysfunction is one of the key mechanisms in DN that causes glomerular injury [26]. A retrospective study demonstrated that anemia was a risk factor for decreased renal function in patients with T2DM with preserved renal function and normal albuminuria, and the trend toward decreased renal function was greater with decreasing Hb levels (OR = 0.69, 95% CI = 0.47–0.99), as demonstrated by the current results. The Hb level test can preliminarily estimate a patient’s renal condition; the lower the Hb level, the higher the CKD stage. Based on the aforementioned evidence, we concluded that the Hb level was significantly correlated with DN and could be used to diagnose DN.

Previous studies have identified several DN-related biomarkers, including eGFR, UACR, advanced glycation end products (AGEs), and so on. Although eGFR can reflect renal function relatively directly, it remains unchanged or slightly increases in the early stages of DN. This indicates that eGFR has obvious limitations for the early identification of DN. Our results revealed that a decrease in Hb level was indicative of early DN [27]. Although UACR is important for diagnosing early DN, approximately 23.3–56.6% of patients with DN exhibit normal urinary albumin levels. Additionally, UACR can be affected by other diseases [28, 29]. Recent studies have demonstrated that the generation of AGEs is an important mechanism for DN occurrence and progression and can be used as a predictor of DN. However, its detection requirements are stricter and are significantly affected by diet [30, 31]. *AGEs are well-known biomarkers of DN*,* and we have previously elucidated the role of AGEs in DN* [32, 33]. Our results revealed that Hb exhibited a slightly higher diagnostic value than eGFR, indicating that Hb could be used as a diagnostic marker for DN. *Although Hb is less effective than UACR in predicting DN*,* Hb detection is cheaper and more convenient.* Hb is a common indicator in routine blood tests, is easy to obtain, and has a relatively stable level. Consequently, it could become a more meaningful predictor of DN.

### Limitations

This study has certain limitations. First, this was a retrospective study, which may have caused selection bias. Second, this was an observational study. It is difficult to determine the causal relationship between Hb level and renal function progression in patients with diabetes. Third, although confounding factors such as age and gender were considered in this study, the effects of medication or treatment on patients were not discussed. Medication or treatment effects may influence DN, and the effects of drugs on DN must be investigated in future studies. *Finally*,* because UACR is not necessary for some patients with end-stage renal disease*,* we only discussed its predictive ability in patients with CKD1–4. As AGEs are recognized biomarkers of DN*,* previous studies by our research team have confirmed their role in DN. Additionally*,* detecting AGEs is complex and expensive. Currently*,* AGEs have not been clinically tested in our research unit*,* and a comparison between Hb and AGEs has not been conducted in this study.*

## Conclusion

The results of this study indicate that low Hb levels are closely associated with DN occurrence, which is an independent risk factor for DN and can be used as a predictive biomarker for DN.

## Electronic supplementary material

Below is the link to the electronic supplementary material.

Supplementary Material 1

Supplementary Material 2
