# Supplementary material for: Correlation analysis between hemoglobin and type 2 diabetic nephropathy: a two-center retrospective study
Source: Acta Diabetol. 2025 Jun 5;62(7):1149–56. doi: 10.1007/s00592-025-02529-9 (PMC12283885; doi:10.1007/s00592-025-02529-9)
Supplement: Supplementary file 1 — Supplementary Material 1 [file 592_2025_2529_MOESM1_ESM.pdf]

Freescience Editorial Team

Certificate of English Editing

Paper Title

Correlation analysis between hemoglobin and type 2 diabetic nephropathy: A Two-center Retrospective Study

Authors

Xiaoling Liu, Ze Zhang, Lu Lin, Jinghui Li, Bende Liu, Huaqian Chen, Junwei Zhou, Pin Chen

This certificate is issued as a confirmation that the paper mentioned above has been proofread and edited for language clarity and grammar by professional editors（Name:William Pat Fong） of our company.

We guarantee that the original message was not distorted, and that the paper is understandable and free of errors assuming that the changes and suggestions given are accepted, and text is not altered without our knowledge.

Date of Editing: 02-12-2025
