# Supplementary material for: Correlation analysis between hemoglobin and type 2 diabetic nephropathy: a two-center retrospective study
Source: Acta Diabetol. 2025 Jun 5;62(7):1149–56. doi: 10.1007/s00592-025-02529-9 (PMC12283885; doi:10.1007/s00592-025-02529-9)
Supplement: Supplementary file 2 — Supplementary Material 2 [file 592_2025_2529_MOESM2_ESM.docx]

**Statements and Declarations**

  The authors declare that they have no conflict of interest.

**Funding:**

This study was supported by the National Natural Science Foundation of China (No. 81870581)

**Disclosures and declarations**

This study has been approved by the Ethics committee of our hospital (Ethics No.:2023-055). This study is a retrospective study and does not involve the privacy information of patients, so it is not necessary to sign the informed consent of patients.
